# Factors Influencing Maternal and Neonatal Mortality in South Asia and Southeast Asia (2010-2023): A Comparative Panel Data Analysis

**DOI:** 10.7759/cureus.115800

**Published:** 2026-09-04

**Authors:** Kratika Shah, Sheetal Shah, Himamshu Soni

**Affiliations:** 1 Science (Biology), Bal Bharati Public School, New Delhi, IND; 2 Obstetrics and Gynaecology, Shah Hospital, Gandhidham, IND; 3 Pathology, Employees' State Insurance Corporation (ESIC) Medical College, Indore, IND

**Keywords:** health determinant, maternal mortality, neonatal mortality, panel data analysis, south and southeast asia

## Abstract

Background

Maternal and neonatal mortality remains a critical public health challenge in low- and middle-income countries, particularly in South Asia and Southeast Asia. Despite progress, regional disparities persist, and the influence of socioeconomic and health system factors varies across contexts. This study aimed to comparatively assess determinants of maternal mortality ratio (MMR) and neonatal mortality rate (NMR) across these regions.

Methods

An ecological longitudinal panel study was conducted using data from the World Bank for 19 countries (2010-2023). Outcomes included MMR and NMR, while explanatory variables were health expenditure, gross domestic product (GDP) per capita, adolescent fertility rate (AFR), anaemia prevalence among pregnant women, and a COVID-19 indicator. Log-transformed variables were analysed using fixed and random effects regression models, with Hausman tests guiding model selection. Stratified and interaction analyses assessed regional differences.

Results

Both regions showed declining mortality trends, though South Asia consistently exhibited higher MMR and NMR. GDP per capita was inversely associated with both outcomes across regions. In South Asia, health expenditure and GDP were protective, while AFR significantly increased both MMR and NMR. In Southeast Asia, anaemia prevalence emerged as the strongest predictor of increased mortality. The COVID-19 period was associated with a significant rise in maternal mortality but not neonatal mortality. Interaction models revealed stronger effects of health expenditure in South Asia. In addition, the association between adolescent fertility and mortality differed by region, being negative in Southeast Asia but positive in South Asia, indicating a reversal in the direction of association across regions.

Conclusion

Maternal and neonatal mortality outcomes in South Asia and Southeast Asia are associated with region-specific socioeconomic, demographic, and health-system factors. Economic development is protective, whereas adolescent fertility and maternal anaemia are key risk factors. Targeted, context-specific interventions focusing on strengthening health systems, reducing adolescent pregnancies, and addressing maternal anaemia are essential for further mortality reduction.

## Introduction

The issues of maternal and neonatal mortality continue to be major public health concerns, especially for low- and middle-income countries, where the burden is very high [1,2]. Although considerable achievements were made under the Millennium Development Goals (MDGs), inequalities still remain between different regions within the Sustainable Development Goals (SDGs) framework [2,3]. Maternal mortality ratio (MMR) and neonatal mortality rate (NMR) are commonly used measures that not only indicate the state of the healthcare systems but also the socio-economic and demographic situation affecting the population health [4,5].

South Asia and Southeast Asia are two regions that are similar in terms of geographical proximity but vary greatly in socio-economic and structural dimensions [6]. South Asia and Southeast Asia were selected for comparison because they collectively bear a substantial burden of maternal and neonatal mortality while encompassing countries with diverse levels of economic development, health-system capacity, demographic characteristics, and reproductive health outcomes. This regional diversity provides an important opportunity to examine whether factors associated with maternal and neonatal mortality differ across contexts. Factors such as differences in the quality of healthcare facilities, execution of healthcare policies, culture, and economic development account for differences in the level of reproductive health between these regions [7]. Various factors have been highlighted as determinants of maternal and neonatal mortality, which include maternal anaemia, healthcare expenditure, economic development, and adolescent fertility rates [8]. For instance, maternal anaemia is linked with poor pregnancy outcomes and neonatal health [9]. On the other hand, increased healthcare expenditure and better economic status are linked with reduced mortality rates [10]. Adolescent fertility is also an important indicator of reproductive risk and access to reproductive health services, both of which are critical in determining outcomes for mothers and newborns [11,12].

These determinants have been widely studied, but it is worth mentioning that their relative influence can vary depending on the regional context. Governance, health system capacity, and social determinants may alter the nature of the relationship among these determinants. Therefore, understanding such contextual differences becomes essential for designing targeted interventions and maximizing policy decisions.

Extensive research has been done on maternal and neonatal mortality, but the evidence is still fragmented. Most studies are country-specific or subnational and focus on individual contexts like Indonesia or a specific province, thus limiting cross-regional generalizability. The regional analyses in South Asia and Southeast Asia have been largely descriptive or policy-oriented, stressing progress and inequalities, and have not applied consistent econometric frameworks. Furthermore, systematic reviews have identified a number of determinants, but the evidence synthesis across different settings is limited and not integrated within a comparative analytical framework. Importantly, few studies employ longitudinal panel data to examine how these determinants operate across countries and over time, and even fewer assess whether such relationships differ between regions [8,10,13-15].

This study aims to compare factors associated with maternal and neonatal mortality between South Asia and Southeast Asia using panel data analysis, with the objective of identifying region-specific associations related to mortality outcomes and generating evidence to support context-sensitive public health strategies.

## Materials and methods

Study design

This study employed an ecological longitudinal panel design using secondary country-level data to examine the determinants of maternal and neonatal mortality across South Asia and Southeast Asia. The panel structure allowed for the analysis of both cross-sectional and temporal variations in mortality outcomes and their associated factors over time, thereby providing a comparative and dynamic assessment of trends and determinants across regions.

Study setting and population

The study included a total of 19 countries categorized into two regions based on geographical classification. South Asia comprised Afghanistan, Bangladesh, Bhutan, India, Maldives, Nepal, Pakistan, and Sri Lanka, while Southeast Asia included Indonesia, Thailand, Vietnam, Philippines, Malaysia, Cambodia, Lao People’s Democratic Republic, Myanmar, Singapore, Timor-Leste, and Brunei Darussalam. These countries were selected based on their regional affiliation and the availability of relevant data for the study variables within the study period. All identified countries met the inclusion criteria and were retained in the final analysis; therefore, no countries were excluded.

Study period

The study period spanned from 2010 to 2023, depending on the availability of data for each country and variable. This timeframe was chosen to capture long-term trends in maternal and neonatal mortality as well as the potential influence of recent global events, including the COVID-19 pandemic.

Data source

All data for this study were obtained from the World Bank's World Development Indicators (WDI) database, a publicly accessible and standardized repository of global development data [16]. Maternal mortality was measured using the modelled MMR indicator (WB_WDI_SH_STA_MMRT), while neonatal mortality (WB_HCP_NEOMORT), current health expenditure (CHE) (WB_WDI_SH_XPD_CHEX_GD_ZS), gross domestic product (GDP) per capita (WB_WDI_NY_GDP_PCAP_CD), adolescent fertility rate (AFR) (WB_WDI_SP_ADO_TFRT), and prevalence of anaemia among pregnant women (WB_WDI_SH_PRG_ANEM) were obtained from their corresponding WDI indicators. The complete list of WDI indicators, codes, and units of measurement is provided in Supplementary Table S1.

The MMR indicator (WB_WDI_SH_STA_MMRT) is a modelled estimate produced through the Maternal Mortality Estimation Inter-Agency Group methodology. Because the estimation framework incorporates socioeconomic covariates, including GDP per capita measured using purchasing power parity (PPP)-related information, and GDP per capita was also included as an explanatory variable in the present analysis, the association between GDP per capita and maternal mortality was interpreted with caution owing to the potential for partial non-independence between the outcome measure and predictor variable.

Study variables

The dependent variables in this study were the MMR, defined as the number of maternal deaths per 100,000 live births, and the NMR, defined as the number of deaths within the first 28 days of life per 1,000 live births. The explanatory variables were selected based on previous evidence identifying socioeconomic, health-system, and maternal risk factors associated with maternal and neonatal mortality [8,10,14]. These included CHE as a percentage of GDP, GDP per capita in current US dollars, AFR measured as births per 1,000 women aged 15-19 years, and the prevalence of anaemia among pregnant women expressed as a percentage. These variables were chosen because they were consistently available across the study countries and time period (2010-2023) and represented key domains of economic development, health-system investment, reproductive risk, and maternal health status. Other established determinants, such as female educational attainment, skilled birth attendance, and healthcare workforce density, were not included because complete and comparable longitudinal data were not available for all countries during the study period.

Additionally, a binary variable representing the COVID-19 pandemic period was included, coded as 1 for the years 2020 to 2022 and 0 for all other years. This period was selected because it encompasses the years during which the pandemic caused the greatest global disruption to healthcare systems, maternal health services, and population health outcomes. Although the timing and magnitude of COVID-19 impacts varied across countries and some effects extended beyond 2022, the 2020-2022 period broadly captures the principal phase of pandemic-related disruptions across South Asia and Southeast Asia.

Data management

The data were extracted and organized in a panel format consisting of country-year observations. Continuous variables were transformed using natural logarithms to improve their distributional properties, reduce heteroscedasticity, and facilitate interpretation of regression coefficients as percentage changes. Data completeness was assessed across all study variables during the data preparation process. No missing values were identified in the final panel dataset used for analysis. Therefore, interpolation, imputation, listwise deletion, and sensitivity analyses related to missing-data handling were not required.

These countries were selected based on their regional affiliation and the availability of relevant data for the study variables within the defined time period. All identified countries met the inclusion criteria and were retained in the final analysis; therefore, no countries were excluded.

Statistical analysis

Descriptive and Exploratory Analysis

All statistical analyses were performed using STATA version 15 (StataCorp LLC, College Station, TX) [17]. Descriptive statistics, including means, standard deviations, skewness, and kurtosis, were computed to summarize the distribution and characteristics of the study variables across countries and regions. Trends in maternal and neonatal mortality over time were examined using graphical representations to illustrate regional patterns.

Descriptive statistics were calculated using all available country-year observations from 2010 to 2023 to summarize the overall characteristics of the panel dataset. These pooled statistics represent average levels across the study-period observations and were not intended to represent baseline or end-of-study values. To additionally assess temporal changes over the study period, regional averages were calculated separately for 2010 and 2023 for MMR, NMR, CHE, GDP per capita, AFR, and prevalence of anaemia among pregnant women (PAP). For each region and year, the reported value represents the arithmetic mean of the country-level observations within that region. The percentage change between 2010 and 2023 was calculated as [(2023 value − 2010 value) / 2010 value] × 100. Temporal comparisons were presented on the original measurement scales to facilitate interpretation, whereas log-transformed variables were used in the regression analyses.

Multicollinearity Diagnosis

Pearson correlation analysis was conducted to assess the relationships between dependent and independent variables. To further evaluate multicollinearity among explanatory variables, variance inflation factors (VIFs) were calculated. VIF values below commonly accepted thresholds were considered indicative of the absence of problematic multicollinearity.

Panel Data Regression Analysis

Panel data regression techniques were employed to evaluate the association between explanatory variables and mortality outcomes. Separate models were specified for maternal mortality and neonatal mortality.

Model 1: MMR as the Dependent Variable

$\ln(MMR_{it}) = \beta_0 + \beta_1 \ln(PAP_{it}) + \beta_2 \ln(CHE_{it}) + \beta_3 \ln(GDP_{it}) + \beta_4 \ln(AFR_{it}) + \mu_i + \varepsilon_{it}$

Where,

$\ln(MMR_{it}) = \text{Natural logarithm of the MMR in country } i \text{ at time } t$

$\ln(PAP_{it}) = \text{Natural logarithm of the Prevalence of anaemia among pregnant women (\%)}$

$\ln(CHE_{it}) = \text{Natural logarithm of the CHE (\% GDP)}$

$\ln(GDP_{it}) = \text{Natural logarithm of the GDP per capita (current US\$)}$

$\ln(AFR_{it}) = \text{Natural logarithm of the AFR}$

$\mu_i = \text{Country-specific effect}$

$\varepsilon_{it} = \text{Error term}$

Model 2: NMR as the Dependent Variable

$\ln(NMR_{it}) = \beta_0 + \beta_1 \ln(PAP_{it}) + \beta_2 \ln(CHE_{it}) + \beta_3 \ln(GDP_{it}) + \beta_4 \ln(AFR_{it}) + \mu_i + \varepsilon_{it}$

Where,

$\ln(NMR_{it}) = \text{Natural logarithm of the Neonatal Mortality Ratio in country } i \text{ at time } t$

$\ln(PAP_{it}) = \text{Natural logarithm of the Prevalence of anaemia among pregnant women (\%)}$

$\ln(CHE_{it}) = \text{Natural logarithm of the CHE (\% GDP)}$

$\ln(GDP_{it}) = \text{Natural logarithm of the GDP per capita (current US\$)}$

$\ln(AFR_{it}) = \text{Natural logarithm of the AFR}$

$\mu_i = \text{Country-specific effect}$

$\varepsilon_{it} = \text{Error term}$

Pooled ordinary least squares (OLS) models with country-clustered robust standard errors were estimated as baseline specifications. Fixed effects (FE) and random effects (RE) models were subsequently estimated to account for unobserved country-specific heterogeneity. The Hausman specification test was used to guide the selection between the FE and RE specifications, with the selected panel model used for the principal analysis. The general form of the regression model included log-transformed dependent and independent variables along with a COVID-19 indicator variable, incorporating both country-specific effects and random error terms.

Panel Data Model Diagnostics and Model Selection

The Hausman specification test was used to determine the appropriate model between FE and RE approaches, with a p-value less than 0.05 indicating preference for the FE model. Standard panel-data diagnostic tests were conducted to assess model assumptions. Heteroscedasticity was evaluated using the modified Wald test for groupwise heteroscedasticity, serial correlation was assessed using the Wooldridge test for autocorrelation in panel data, and cross-sectional dependence was examined using the Pesaran cross-sectional dependence (CD) test.

Stratified and Interaction Analysis

Stratified analyses were conducted separately for South Asia and Southeast Asia to explore region-specific associations. In addition, pooled models with interaction terms between region and explanatory variables were estimated to assess differences in effect sizes between the two regions.

Statistical Significance

A p-value of less than 0.05 was considered statistically significant for all analyses.

Ethical considerations

This study was based entirely on publicly available secondary data and did not involve human participants directly. Therefore, ethical approval was not required. All data were used in accordance with the World Bank’s data usage policies, and appropriate acknowledgements were made to the data source.

## Results

Countries included in the panel dataset are shown in Table 1 along with their respective country codes. The panel dataset comprised 19 countries from South Asia and Southeast Asia, including eight countries from South Asia and 11 countries from Southeast Asia. These countries were selected based on the availability of complete data for the study variables over the study period.

**Table 1:** Countries included in the panel analysis. MMR in the country code column refers to the ISO 3166-1 alpha-3 code for Myanmar, whereas MMR elsewhere in the manuscript denotes maternal mortality ratio.

Country	Country code
South Asia (n = 8)	
Afghanistan	AFG
Bangladesh	BGD
Bhutan	BTN
India	IND
Maldives	MDV
Nepal	NPL
Pakistan	PAK
Sri Lanka	LKA
Southeast Asia (n = 11)	
Brunei Darussalam	BRN
Cambodia	KHM
Indonesia	IDN
Lao People's Democratic Republic	LAO
Malaysia	MYS
Myanmar	MMR
Philippines	PHL
Singapore	SGP
Thailand	THA
Timor-Leste	TLS
Vietnam	VNM

Table 2 presents the definitions of the study variables in the panel dataset. The analysis incorporated two outcome variables: the natural logarithm of the MMR and the neonatal mortality rate (ln NMR). Explanatory variables included the natural logarithms of CHE as a percentage of GDP (ln_CHE), GDP per capita (ln_GDP), AFR (ln_AFR), and the prevalence of anaemia among pregnant women (ln_PAP). A binary COVID-19 indicator variable was additionally created to represent the peak pandemic years (2020-2022), with all other years coded as zero. Logarithmic transformation was applied to all continuous variables to improve normality, reduce heteroscedasticity, and facilitate interpretation of regression coefficients.

**Table 2:** Definitions of study variables. MMR: maternal mortality ratio; NMR: neonatal mortality rate; CHE: current health expenditure; GDP: gross domestic product; AFR: adolescent fertility rate.

Variable	Description
ln_MMR	Natural logarithm of MMR (per 100,000 live births)
ln_NMR	Natural logarithm of NMR (per 1,000 live births)
ln_CHE	Natural logarithm of CHE (% of GDP)
ln_GDP	Natural logarithm of GDP per capita (current US$)
ln_AFR	Natural logarithm of AFR (births per 1,000 women aged 15–19 years)
ln_PAP	Natural logarithm of the prevalence of anaemia among pregnant women (%)
COVID	A binary indicator variable coded as 1 for the COVID-19 pandemic years (2020-2022) and 0 otherwise.

The descriptive statistics of maternal mortality, neonatal mortality, and selected health indicators across South Asian and Southeast Asian countries from 2010 to 2023 are presented in Table 3. These statistics represent average levels across the study-period observations and are intended to summarize the overall characteristics of the panel dataset. In South Asia, Afghanistan reported the highest mean maternal mortality (ln_MMR = 6.54), while Sri Lanka recorded the lowest (3.13). Pakistan exhibited the highest neonatal mortality (ln_NMR = 3.78), whereas Sri Lanka had the lowest (1.64). In Southeast Asia, Timor-Leste showed the highest maternal mortality (ln_MMR = 5.68), while Singapore reported the lowest (2.32). Neonatal mortality was highest in Timor-Leste (ln_NMR = 3.20) and lowest in Singapore (0.03). Overall, South Asia had higher average maternal mortality (4.78 vs. 4.39), neonatal mortality (2.87 vs. 2.26), adolescent fertility (3.48 vs. 3.25), and prevalence of anaemia among pregnant women (3.63 vs. 3.39) than Southeast Asia. Conversely, Southeast Asia demonstrated a higher average GDP per capita (8.46 vs. 7.57). The skewness and kurtosis statistics indicated generally acceptable distributional properties across variables.

**Table 3:** Descriptive statistics of maternal mortality, neonatal mortality, and health system indicators in South Asian and Southeast Asian countries (2010-2023). SD: standard deviation; SK: skewness; KU: kurtosis.

	ln_MMR			ln_NMR			ln_CHE			ln_GDP			ln_AFR				ln_PAP		
	Mean (SD)	SK	KU	Mean (SD)	SK	KU	Mean (SD)	SK	KU	Mean (SD)	SK	KU		Mean (SD)	SK	KU	Mean (SD)	SK	KU
South Asia																			
AFG	6.54 (0.16)	0.13	2.12	3.7 (0.11)	0.10	1.81	2.51 (0.34)	0.43	2.10	6.24 (0.2)	-0.74	2.52		4.37 (0.16)	0.44	1.97	3.49 (0.03)	0.29	1.83
BGD	5.24 (0.26)	0.34	2.28	3.1 (0.17)	0.17	1.57	0.82 (0.04)	-0.44	2.19	7.32 (0.46)	-0.23	1.50		4.47 (0.15)	0.45	1.92	3.71 (0.04)	0.23	1.72
BTN	4.17 (0.27)	0.55	2.01	2.79 (0.17)	0.20	1.84	1.25 (0.14)	1.04	2.77	8.05 (0.14)	-0.21	1.88		2.66 (0.48)	0.39	1.62	3.53 (0.03)	0.34	1.92
IND	4.83 (0.26)	0.07	1.97	3.17 (0.2)	-0.18	1.79	1.2 (0.08)	-0.52	2.43	7.49 (0.2)	0.20	1.76		3.03 (0.46)	0.64	1.56	3.87 (0.03)	0.26	1.71
MDV	3.76 (0.16)	0.03	3.02	1.7 (0.19)	-0.20	2.25	2.15 (0.12)	0.80	3.15	9.15 (0.2)	-0.10	1.59		2.26 (0.42)	0.03	1.51	3.8 (0.05)	0.18	1.81
NPL	5.32 (0.26)	0.08	1.65	3.08 (0.17)	-0.07	1.72	1.62 (0.13)	0.65	2.27	6.89 (0.26)	-0.26	2.14		4.33 (0.08)	-0.20	1.51	3.62 (0.04)	0.11	1.70
PAK	5.27 (0.14)	0.02	1.92	3.78 (0.09)	-0.13	1.78	0.96 (0.10)	-0.14	1.77	7.19 (0.13)	-0.74	2.99		3.86 (0.09)	0.04	2.20	3.76 (0.06)	0.41	1.77
LKA	3.13 (0.15)	1.19	5.15	1.64 (0.14)	-0.07	1.82	1.32 (0.11)	1.74	5.90	8.23 (0.13)	-0.89	2.96		2.85 (0.14)	0.66	1.83	3.26 (0.07)	0.56	2.04
Total	4.78 (1.02)	0.00	2.27	2.87 (0.78)	-0.59	2.07	1.48 (0.57)	0.89	2.97	7.57 (0.87)	0.26	2.47		3.48 (0.87)	-0.29	1.71	3.63 (0.19)	0.62	2.67
Southeast Asia																			
BRN	3.73 (0.22)	3.12	11.21	1.61 (0.03)	0.27	1.37	0.76 (0.12)	-0.27	1.64	10.44 (0.2)	0.43	1.85		2.41 (0.22)	0.15	1.62	3.09 (0.04)	0.55	2.04
KHM	5.15 (0.15)	0.63	2.6	2.74 (0.19)	0.18	1.81	1.6 (0.13)	0.59	1.96	7.42 (0.30)	-0.44	1.91		3.93 (0.05)	0.00	1.75	3.8 (0.05)	0.05	1.73
IDN	5.24 (0.17)	0.19	2.02	2.59 (0.16)	0.12	1.78	1.09 (0.09)	1.52	4.68	8.24 (0.13)	0.48	2.57		3.54 (0.22)	0.08	1.42	3.41 (0.07)	0.14	1.72
LAO	5.14 (0.31)	0.48	2.11	3.17 (0.12)	0.17	1.81	0.81 (0.19)	-1.35	5.12	7.61 (0.26)	-1.01	3.04		4.44 (0.03)	0.68	2.4	3.5 (0.07)	0.00	1.75
MYS	3.43 (0.17)	2.33	7.56	1.44 (0.02)	-0.26	1.86	1.31 (0.08)	-0.17	2.86	9.25 (0.08)	-0.43	2.39		2.27 (0.32)	-0.36	1.69	3.43 (0.04)	0.33	1.91
MMR	5.38 (0.11)	0.06	1.78	3.18 (0.10)	0.06	1.78	1.33 (0.35)	-0.99	2.56	7.12 (0.09)	-0.02	3.79		3.54 (0.02)	-0.64	2.5	3.78 (0.03)	1.02	2.72
PHL	4.63 (0.16)	1.02	3.86	2.63 (0.03)	0.21	1.55	1.47 (0.14)	0.99	2.71	8 (0.16)	-0.5	2.65		3.82 (0.25)	-0.31	1.49	3.37 (0.11)	0.27	1.86
SGP	2.32 (0.45)	1.59	4.89	0.03 (0.08)	-0.78	1.99	1.41 (0.17)	0.05	2.51	11.05 (0.19)	0.7	2.54		0.99 (0.24)	0.76	1.98	2.86 (0.03)	0.26	1.73
THA	3.64 (0.31)	3.24	11.7	1.84 (0.13)	0.1	1.83	1.38 (0.14)	0.96	2.79	8.75 (0.13)	-0.18	1.85		3.67 (0.29)	-0.24	1.4	3.29 (0.07)	0.44	1.85
TLS	5.68 (0.23)	0.17	2.26	3.2 (0.07)	0.17	2.03	1.94 (0.18	-0.45	3.2	7.22 (0.32)	0.63	3.06		3.55 (0.18)	0.19	1.61	3.46 (0.03)	0.43	1.93
VNM	4.01 (0.08)	0.2	1.71	2.4 (0.04)	-0.15	1.93	1.56 (0.05)	-0.35	1.68	7.96 (0.28)	-0.24	2.13		3.62 (0.07)	0.52	1.76	3.27 (0.02)	0.77	2.03
Total	4.39 (1.03)	0.58	2.52	2.26 (0.93)	-1.06	3.48	1.33 (0.37)	-0.25	2.83	8.46 (1.26)	0.86	2.62		3.25 (0.95)	-1.19	3.62	3.39 (0.26)	0.21	2.72

Changes in maternal mortality, neonatal mortality, and associated health and socioeconomic indicators between 2010 and 2023 are presented in Supplementary Table S2. Both regions experienced substantial reductions in maternal and neonatal mortality over the study period, accompanied by increases in GDP per capita and CHE and declines in adolescent fertility and prevalence of anaemia among pregnant women.

Trends in maternal mortality and neonatal mortality are shown in Figure 1 for the years 2010 to 2023 for the countries of South Asia and Southeast Asia. Maternal mortality rates in both regions over time decreased, although South Asia showed consistently higher rates of maternal mortality than Southeast Asia. However, both regions experienced a temporary increase in maternal mortality rates in 2020-2022 during the COVID-19 pandemic (2020-2022), after which rates returned to their declining trajectory (i.e., there was a decrease) in 2023.

**Figure 1:**
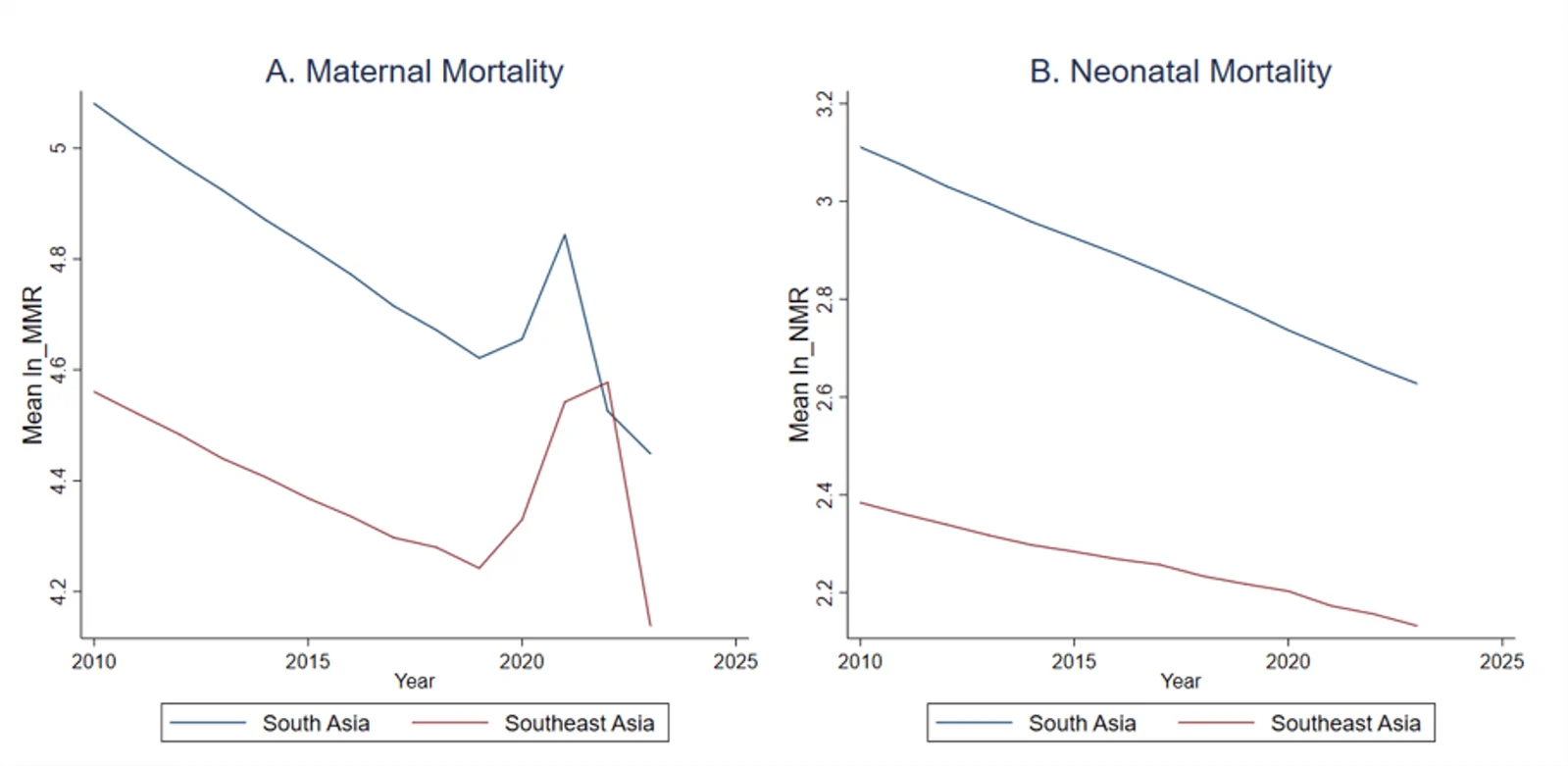
Comparative trends in maternal and neonatal mortality across South Asia and Southeast Asia (2010-2023).

Neonatal mortality showed a steady downward trend throughout the study period in both regions, with no evident increase during the pandemic years. South Asia consistently had higher neonatal mortality than Southeast Asia, although the gap gradually narrowed over time.

Overall, the findings indicate sustained improvements in maternal and neonatal health outcomes, while the COVID-19 period appears to have had a greater impact on maternal mortality than neonatal mortality.

Table 4 presents the Pearson correlation coefficients among maternal mortality, neonatal mortality, and explanatory variables in South Asia and Southeast Asia. In both regions, maternal mortality (ln_MMR) was strongly and positively correlated with neonatal mortality (South Asia: r = 0.890, p < 0.001; Southeast Asia: r = 0.934, p < 0.001). GDP per capita (ln_GDP) demonstrated strong negative correlations with both maternal and neonatal mortality, whereas AFR (ln_AFR) showed strong positive correlations with both outcomes. The prevalence of anaemia among pregnant women (ln_PAP) was positively associated with maternal and neonatal mortality in both regions, with stronger correlations observed in Southeast Asia. CHE exhibited weak or non-significant correlations with mortality indicators. No correlation coefficient exceeded 0.95, indicating the absence of severe multicollinearity among the explanatory variables.

**Table 4:** Pearson correlation matrix of maternal mortality, neonatal mortality, and explanatory variables across South Asia and Southeast Asia. * p-value < 0.05, ** p-value < 0.01, and *** p-value < 0.001.

	ln_MMR	ln_NMR	ln_CHE	ln_GDP	ln_AFR	ln_PAP
	South Asia					
ln_MMR	1.000					
ln_NMR	0.890***	1.000				
ln_CHE	0.207*	-0.135	1.000			
ln_GDP	-0.884***	-0.851***	-0.069	1.000		
ln_AFR	0.810***	0.726***	-0.080	-0.846***	1.000	
ln_PAP	0.261**	0.343***	-0.191*	0.022	0.050	1.000
	Southeast Asia					
ln_MMR	1.000					
ln_NMR	0.934***	1.000				
ln_CHE	0.138	0.101	1.000			
ln_GDP	-0.865***	-0.934***	-0.321***	1.000		
ln_AFR	0.766***	0.891***	0.011	-0.860***	1.000	
ln_PAP	0.773***	0.795***	0.140	-0.849***	0.712***	1.000

VIFs for explanatory variables were also analysed and presented in Supplementary Table S3. The VIF scores were observed in the range of 1.31-5.94, with the average VIF being 3.30. The maximum VIF score was observed for ln_GDP (5.94), followed by ln_AFR (3.95), ln_PAP (2.00) and ln_CHE (1.31). Overall, it indicates that there is no severe multicollinearity among the explanatory variables.

In the initial step, pooled OLS models using robust standard errors were calculated in order to be able to compare them to the panel models and those data are presented in Supplementary Table S4. The results of pooled OLS showed variations in magnitude and direction of association in some estimates relative to the succeeding FE estimates, suggesting the importance of unobserved country-specific heterogeneity. Therefore, in the next stage, FE and RE models were used as the principal panel-data specifications.

Table 5 presents the panel regression results examining factors associated with maternal mortality and neonatal mortality in South Asia and Southeast Asia. Based on the Hausman test, the FE model was selected for maternal mortality in South Asia and neonatal mortality in Southeast Asia, whereas the RE model was preferred for maternal mortality in Southeast Asia. For neonatal mortality in South Asia, the Hausman test was borderline (χ² = 9.38, p = 0.052). Although the null hypothesis of the Hausman test was not rejected at the 5% level, the FE model was retained because the p-value was marginal and fixed effects provide more robust estimates in the presence of potential correlation between unobserved country-specific characteristics and the explanatory variables. This approach also ensured methodological consistency with the pooled FE analysis presented in Table 6. Detailed model-diagnostic results, including tests for heteroscedasticity, serial correlation, and cross-sectional dependence, are provided in Supplementary Table S5. Given the detected error-structure characteristics, Driscoll-Kraay standard errors were used for the final FE estimates, while country-clustered robust standard errors were used for the RE specification.

**Table 5:** Panel regression analysis of factors associated with maternal mortality and neonatal mortality in South Asia and Southeast Asia. FE: fixed effects model; RE: random effects model; SE: standard error; SE^a^: Driscoll–Kraay standard errors; SE^b^: robust standard error; ^†^ Hausman test-preferred specification. * p-value < 0.05, ** p-value < 0.01, and *** p-value < 0.001. Fixed-effects models were selected when the Hausman test was significant (p < 0.05). For South Asia neonatal mortality (p = 0.052), the fixed-effects model was retained because the Hausman result was borderline, and the FE specification allows potential correlation between unobserved country-specific effects and the explanatory variables while maintaining consistency with the pooled FE analysis.

Variable	ln_MMR				ln_NMR			
	South Asia		Southeast Asia		South Asia		Southeast Asia	
	FE^†^	RE	FE	RE^†^	FE^†^	RE	FE^†^	RE
	β (SE^a^)	β (SE)	β (SE)	β (SE^b^)	β (SE^a^)	β (SE)	β (SE^a^)	β (SE)
ln_CHE	-0.428*** (0.095)	-0.307** (0.098)	0.135 (0.113)	0.114 (0.139)	-0.146** (0.040)	-0.152*** (0.034)	-0.043 (0.028)	-0.044 (0.041)
ln_GDP	-0.452*** (0.040)	-0.467*** (0.070)	-0.271* (0.105)	-0.361** (0.134)	-0.196*** (0.012)	-0.206*** (0.025)	-0.230*** (0.0.124)	-0.288*** (0.037)
ln_AFR	0.238*** (0.028)	0.263*** (0.058)	-0.334* (0.144)	-0.181 (0.182)	0.193*** (0.032)	0.195*** (0.019)	-0.086** (0.026)	-0.014 (0.050)
ln_PAP	0.793 (0.461)	0.839 (0.394)	2.321*** (0.559)	1.820* (0.844)	1.424*** (0.124)	1.373*** (0.157)	0.797*** (0.055)	0.633*** (0.194)
COVID	0.124* (0.047)	0.114** (0.038)	0.237*** (0.049)	0.258* (0.106)	-0.016 (0.021)	-0.016 (0.012)	-0.017 (0.009)	0.001 (0.018)
Hausman test	χ^2^ = 20.86, p-value < 0.001		χ^2^ = 6.45, p-value = 0.168		χ^2^ = 9.38, p-value = 0.0523		χ^2^ = 106.87, p-value < 0.001	

**Table 6:** Pooled FE panel regression with regional interaction effects for maternal and neonatal mortality in South Asia and Southeast Asia (2010-2023). * p-value < 0.05, ** p-value < 0.01, and *** p-value < 0.001. Southeast Asia served as the reference category (South Asia = 1, Southeast Asia = 0). Main effects represent estimates for Southeast Asia, while interaction terms (South Asia × Predictor) indicate the difference in the estimated association between South Asia and Southeast Asia. Region-specific associations for South Asia are obtained by adding the corresponding main-effect and interaction coefficients.

Variable	ln_MMR		ln_NMR	
	β (SE)	p-value	β (SE)	p-value
Main effects (Southeast Asia)				
ln_CHE	0.135 (0.097)	0.163	-0.043 (0.033)	0.187
ln_GDP	-0.271 (0.090)	0.003**	-0.230 (0.030)	<0.001***
ln_AFR	-0.334 (0.123)	0.007**	-0.086 (0.041)	0.039*
ln_PAP	2.321 (0.477)	<0.001***	0.797 (0.161)	<0.001***
COVID	0.237(0.042)	<0.001***	-0.017(0.014)	0.233
Interaction effects (South Asia × Predictor)				
South Asia × ln_CHE	-0.563 (0.175)	0.001***	-0.103 (0.059)	0.084
South Asia × ln_GDP	-0.181 (0.138)	0.190	0.034 (0.047)	0.468
South Asia × ln_AFR	0.572 (0.147)	<0.001***	0.279 (0.050)	<0.001***
South Asia × ln_PAP	-1.528 (0.832)	0.068	0.627 (0.281)	0.027*
South Asia × COVID	-0.113(0.067)	0.093	0.0005(0.023)	0.984

In South Asia, maternal mortality was significantly associated with lower health expenditure (β = -0.428, p < 0.001), higher GDP per capita (β = -0.452, p < 0.001), and higher AFRs (β = 0.238, p < 0.001). The COVID-19 period was associated with a significant increase in maternal mortality (β = 0.124, p <0.05). For neonatal mortality, health expenditure (β = -0.146, p < 0.01) and GDP (β = -0.196, p < 0.001) per capita were negatively associated with mortality, whereas AFR (β = 0.193, p < 0.001) and anaemia prevalence among pregnant women were positively associated with neonatal mortality (β = 1.424, p < 0.001). The COVID-19 indicator was not statistically significant.

In Southeast Asia, maternal mortality was negatively associated with GDP per capita (β = -0.361, p < 0.01) and positively associated with anaemia prevalence among pregnant women (β = 1.820, p < 0.05). The COVID-19 period was also associated with increased maternal mortality (β = 0.258, p < 0.05). For neonatal mortality, GDP per capita was negatively associated (β = -0.230, p < 0.001), whereas AFRs (β = 0.086, p < 0.01) and anaemia prevalence among pregnant women (β = 0.797, p < 0.001) were positively associated. Neither health expenditure nor the COVID-19 indicator showed a significant association with neonatal mortality in Southeast Asia.

The pooled FE panel regression results examining the associations of health system and demographic factors with maternal mortality (ln_MMR) and neonatal mortality (ln_NMR) across South and Southeast Asia, including regional interaction effects, are stated in Table 6.

For maternal mortality, in Southeast Asia (reference region), higher GDP per capita was associated with lower maternal mortality (β = -0.271, p = 0.003), while higher prevalence of anaemia among pregnant women was associated with increased maternal mortality (β = 2.321, p < 0.001). The COVID-19 period was also associated with a significant increase in maternal mortality (β = 0.237, p < 0.001). Significant interaction effects indicated that the protective effect of health expenditure was stronger in South Asia than in Southeast Asia (South Asia × ln_CHE: β = -0.563, p = 0.001). Importantly, the association between adolescent fertility and maternal mortality differed in direction between the two regions. In Southeast Asia, adolescent fertility was negatively associated with maternal mortality (β = −0.334, p = 0.007), whereas the positive interaction term (South Asia × ln_AFR: β = 0.572, p < 0.001) resulted in a positive region-specific association in South Asia (approximately β = 0.238). Thus, the regional interaction represents a reversal in the direction of the association, from negative in Southeast Asia to positive in South Asia. No significant regional differences were observed for GDP, anaemia prevalence, or COVID-19 effects.

For neonatal mortality, higher GDP per capita was associated with lower neonatal mortality (β = -0.230, p < 0.001), whereas higher prevalence of anaemia among pregnant women was associated with increased neonatal mortality (β = 0.797, p < 0.001). The COVID-19 variable was not significantly associated with neonatal mortality. Adolescent fertility was negatively associated with neonatal mortality in Southeast Asia (β = −0.086, p = 0.039). However, the significant positive interaction between South Asia and adolescent fertility (β = 0.279, p < 0.001) resulted in a positive region-specific association in South Asia (approximately β = 0.193). Thus, as with maternal mortality, the association between adolescent fertility and neonatal mortality reversed direction between the two regions. Additionally, the positive association between anaemia prevalence and neonatal mortality was significantly greater in South Asia than in Southeast Asia (β = 0.627, p = 0.027). No significant regional differences were observed for health expenditure, GDP, or COVID-19 effects.

Overall, GDP per capita consistently demonstrated a protective association with both maternal and neonatal mortality, while anaemia prevalence among pregnant women emerged as an important risk factor. The COVID-19 period was associated with increased maternal mortality but showed no significant impact on neonatal mortality. Regional differences were primarily observed for health expenditure and AFR. For adolescent fertility, the direction of association differed between regions, with negative associations in Southeast Asia and positive associations in South Asia for both maternal and neonatal mortality.

## Discussion

This study provides a detailed comparative investigation of the dynamics of maternal and neonatal mortality in South Asia and Southeast Asia between 2010 and 2023, employing the techniques of panel data research. According to the results, there is a stable decrease in MMR and NMR in both regions, though South Asia is still under a greater burden than Southeast Asia. Importantly, there was a temporary increase in maternal mortality associated with the COVID-19 pandemic in 2020-2022, whereas neonatal mortality remained largely unaffected.

GDP per capita appeared to be a solid protective factor for both regions, showing significant negative associations with both maternal and neonatal mortality. At the same time, anaemia in pregnancy and AFR turned out to be significant risk factors, though their influence varied depending on the region under consideration. There was a greater protective impact of health expenditure in South Asia, especially in terms of maternal mortality, whereas its effect was lower in Southeast Asia.

Most importantly, the results confirmed the existence of regional heterogeneity. The association between adolescent fertility and both maternal and neonatal mortality differed in direction between the two regions. In South Asia, higher adolescent fertility was positively associated with maternal and neonatal mortality, whereas in Southeast Asia, the corresponding associations were negative. Therefore, the interaction analysis indicates a reversal in the direction of association rather than merely a stronger positive association in South Asia. Anaemia was more strongly associated with MMR in Southeast Asia. These findings highlight the importance of context-specific determinants and underscore the need for tailored public health strategies.

The observed regional heterogeneity suggests that the relationships between socioeconomic and health-system determinants and mortality outcomes are context-dependent rather than uniform across settings. While GDP per capita demonstrated a consistent protective association in both regions, the magnitude and significance of other determinants varied considerably. This pattern may reflect differences in health-system organization, healthcare accessibility, socioeconomic development, and demographic transitions between South Asia and Southeast Asia. Economic development may be associated with maternal and neonatal outcomes through multiple pathways, including improved healthcare access, stronger health systems, and better living conditions [8,15,18,19].

The contrasting associations observed for adolescent fertility were particularly noteworthy. In South Asia, higher adolescent fertility was associated with increased maternal and neonatal mortality, consistent with the established biological and social risks of adolescent pregnancy [20,21]. In contrast, the negative associations observed in Southeast Asia suggest that adolescent fertility may be acting as a marker of broader contextual characteristics rather than representing a direct protective factor. These findings may reflect differences in reproductive health services, healthcare utilization, demographic transitions, or unmeasured social and health-system factors not captured in the present analysis [8,14]. Given the ecological nature of this study, the findings should be interpreted as evidence of regional variation in association rather than evidence of causality.

Moreover, the importance of maternal anaemia, especially in Southeast Asia, confirms previous findings which associate anaemia with poor outcomes of pregnancy, such as complications for mothers and illnesses among newborns, possibly because of differences in nutrition and the response of health systems [22]. The difference in the impact of health expenditure across the two regions further underscores the fact that healthcare expenditure might yield more benefits in South Asia because of the current gaps in access and infrastructure. The observed association between the COVID-19 period and increased maternal mortality may reflect disruptions in antenatal care, skilled birth attendance, and emergency obstetric services [23].

The findings should primarily be interpreted as evidence of the importance of socioeconomic and maternal-health factors in shaping mortality patterns at the population level. The observed associations suggest that adolescent fertility, maternal anaemia, economic development, and health-system investment remain relevant dimensions for maternal and neonatal health policy [24-28]. However, because the present study did not directly evaluate specific clinical interventions, healthcare programmes, or policy initiatives, the results should not be interpreted as evidence regarding the effectiveness of particular interventions. Rather, they identify broad determinants that may warrant consideration when designing region-specific maternal and neonatal health strategies.

The implications drawn from the findings of the research study will be useful in ensuring that policies made to cut down on maternal mortality and neonatal mortality rates are implemented effectively. Effective budgeting of healthcare spending will be necessary, especially in South Asia, since developing more health infrastructure facilities, more healthcare professionals, and good-quality healthcare services can contribute greatly towards better results [27]. Broader economic development strategies that reduce poverty and improve living conditions can also indirectly enhance maternal and neonatal health outcomes [28]. Adolescent fertility needs to be handled through good reproductive health education, contraceptive availability, and policies that discourage early marriages [29]. Increasing nutritional healthcare programs, especially the control of maternal anaemia, is also very important [30].

Although this study offers important information on the factors that influence maternal and neonatal mortality rates, there are many aspects that call for further investigation. Future research should include some more health system variables, including those that measure quality of care. Moreover, studies at subnational levels will help reveal any disparities among countries that might not be evident at the national level. In addition, using longitudinal and causal modelling techniques can assist in understanding how certain socio-economic and health system variables affect mortality rates. It is equally important to conduct an assessment of the impact of certain health policies and interventions aimed at reducing mortality.

Strengths and limitations

This research possesses a number of major strengths that increase the scientific rigour and significance of the work. The study enables the assessment of both temporal trends and cross-country comparisons across 19 countries in South Asia and Southeast Asia. Moreover, the employment of more advanced methods of statistical modelling, such as FE and RE models, increases the reliability of the obtained associations. Finally, the consideration of regional interaction effects provides insight into region-specific associations of key determinants with maternal and neonatal mortality.

However, this research is associated with a number of limitations that should be taken into account. The use of country-level data in this ecological study design does not permit inferences at the individual level and therefore may be subject to ecological fallacy. While the FE models utilize within-country variation over time and account for time-invariant country-specific characteristics, the analysis remains based on aggregate national data and cannot determine individual-level relationships between exposures and outcomes. Moreover, since the study uses secondary data, some problems such as possible measurement errors or inconsistencies in the data can emerge. Furthermore, the COVID-19 indicator was based on a common period definition (2020-2022) applied across all countries and may not fully reflect differences in the timing, duration, and intensity of pandemic-related disruptions at the national level. Consequently, the estimated COVID-19 effects should be interpreted as average regional impacts rather than country-specific effects. Cross-sectional dependence was detected in certain regional panels. While robust estimation methods were applied to mitigate the effects of heteroscedasticity and serial correlation, the RE model for Southeast Asia MMR relied on country-clustered standard errors, which account for within-country dependence but do not fully address cross-sectional dependence. Therefore, the corresponding estimates should be interpreted cautiously.

A further limitation concerns the use of the modelled MMR from the WDI. The MMR estimation methodology incorporates GDP measured using purchasing power parities among the socioeconomic inputs used to generate the modelled estimates. GDP per capita was also included as an explanatory variable in the present analysis. Although the GDP measure used in this study was obtained separately from the WDI and expressed as GDP per capita in current US dollars, the use of GDP-related information in the underlying MMR estimation model introduces potential non-independence between the modelled outcome and the GDP predictor. Therefore, the observed GDP-MMR association should be interpreted cautiously and should not be interpreted as evidence of an independent causal effect. The study also includes a limited range of explanatory variables. Several well-established determinants of maternal and neonatal mortality, including female educational attainment, skilled birth attendance, physician density, midwife density, and broader indicators of healthcare access and quality, were not included because complete and comparable longitudinal data were unavailable across all countries and years studied. The omission of these variables may have resulted in residual confounding and may partially explain some of the regional differences observed in the analysis.

The observational design prevents establishing cause-and-effect relationships; therefore, the observed associations should be interpreted with caution. Additionally, year fixed effects were not included in the final regression models. Consequently, the estimated associations may partially reflect unobserved temporal factors that affected all countries simultaneously during the study period, and the findings should therefore be interpreted as associations rather than causal effects.

## Conclusions

Overall, this study found that several socio-economic and health-related factors were associated with maternal and neonatal mortality rates in South Asia and Southeast Asia. However, despite overall improvements, there continue to be disparities in these regions. GDP per capita consistently demonstrated a protective association with maternal and neonatal mortality across both regions, whereas maternal anaemia was associated with increased mortality. The association between adolescent fertility and mortality differed markedly between regions, showing positive associations in South Asia but negative associations in Southeast Asia. These findings highlight important regional heterogeneity in the determinants of maternal and neonatal mortality and underscore the need for context-specific public health strategies aimed at improving maternal health outcomes. The research has also revealed the vulnerability of maternal healthcare systems during the COVID-19 pandemic. Overall, reducing mortality in these regions requires context-specific, integrated strategies focused on strengthening health systems, improving maternal nutrition, addressing adolescent pregnancy, and ensuring continuity of care during public health crises to achieve sustained progress towards global health targets.

## Appendices

Supplementary Table S1

**Table 7:** Description of study variables and associated World Development Indicator (WDI) measures. GDP: gross domestic product.

Study variable	WDI indicator	WDI code	Unit
Maternal mortality ratio	Maternal mortality ratio (modelled estimate, per 100,000 live births)	WB_WDI_SH_STA_MMRT	Per 100,000 live births
Neonatal mortality rate	Neonatal Mortality rate (per 1,000 live births)	WB_HCP_NEOMORT	Per 1,000 live births
Current health expenditure	Current health expenditure (% of GDP)	WB_WDI_SH_XPD_CHEX_GD_ZS	% of GDP
GDP per capita	GDP per capita (current US$)	WB_WDI_NY_GDP_PCAP_CD	Current US$
Adolescent fertility rate	Adolescent fertility rate (births per 1,000 women ages 15-19)	WB_WDI_SP_ADO_TFRT	Births per 1,000 women
Prevalence of anaemia among pregnant women	Prevalence of anaemia among pregnant women (%)	WB_WDI_SH_PRG_ANEM	%
COVID-19 period	Study-derived binary indicator	Not a WDI indicator	0/1

Supplementary Table S2

**Table 8:** Comparison of regional average values for study variables in South Asia and Southeast Asia (2010 and 2023). Values represent regional averages across countries within each region for 2010 and 2023. Percentage change was calculated as [((2023 value − 2010 value) / 2010 value) × 100]. Negative values indicate reductions between 2010 and 2023. Values are presented on their original measurement scales to facilitate interpretation of baseline-to-end-of-study changes. Log-transformed variables were used in the regression analyses. GDP: gross domestic product.

Variable	South Asia (2010)	South Asia (2023)	% Change	Southeast Asia (2010)	Southeast Asia (2023)	% Change
Maternal mortality ratio (MMR)	259.38	138.75	-46.5%	161.27	90.91	-43.6%
Neonatal mortality rate (NMR)	27.79	18.05	-35.0%	15.20	11.32	-25.5%
Current health expenditure (% GDP)	4.56	5.82	27.6%	3.73	4.33	16.0%
GDP per capita (US$)	2,081.96	3,556.92	70.8%	9,714.64	14,283.63	47.0%
Adolescent fertility rate (per 1,000 women aged 15-19 years)	58.59	36.18	-38.2%	40.72	29.53	-27.5%
Prevalence of anaemia among pregnant women (%)	40.78	35.99	-11.7%	33.07	28.37	-14.2%

Supplementary Table S3

**Table 9:** Variance inflation factor (VIF) analysis for the assessment of multicollinearity among independent variables.

Variable	VIF	1/VIF
ln_GDP	5.94	0.17
ln_AFR	3.95	0.25
ln_PAP	2.00	0.50
ln_CHE	1.31	0.77
Mean VIF	3.3	-

Supplementary Table S4

**Table 10:** Baseline pooled ordinary least squares regression estimates. * p-value < 0.05, ** p-value < 0.01, and *** p-value < 0.001. MMR: maternal mortality ratio; NMR: neonatal mortality rate.

Variable	MMR South Asia	MMR Southeast Asia	NMR South Asia	NMR Southeast Asia
	β (SE)			
ln_CHE	0.430 (0.105)**	−0.539 (0.339)	−0.200 (0.122)	−0.508 (0.200)*
ln_GDP	−0.758 (0.117)***	−0.772 (0.312)*	−0.874 (0.116)***	−0.695 (0.181)**
ln_AFR	0.319 (0.158)	−0.091 (0.248)	−0.119 (0.144)	0.130 (0.146)
ln_PAP	1.659 (0.443)**	0.259 (0.883)	1.382 (0.467)*	−0.245 (0.393)
COVID	0.088 (0.131)	0.323 (0.128)*	−0.009 (0.080)	0.105 (0.043)*
Constant	2.735 (2.079)	10.998 (5.742)	5.185 (1.913)*	9.198 (3.032)*
Observations	112	154	112	154
R²	0.920	0.790	0.877	0.925
F-statistic	136.61***	49.51***	50.27***	61.08***

Supplementary Table S5

**Table 11:** Diagnostic evaluation of panel regression assumptions across South Asian and Southeast Asian regions. * p-value < 0.05, ** p-value < 0.01, and *** p-value < 0.001. MMR: maternal mortality ratio; NMR: neonatal mortality rate; CD: cross-sectional dependence.

Outcome	Region	Heteroscedasticity: Modified Wald test	Serial correlation: Wooldridge test	Cross-sectional dependence: Pesaran CD test	Overall conclusion
MMR	South Asia	χ²(8) = 139.57***	F(1,7) = 12.490**	6.365***	Heteroscedasticity, serial correlation and cross-sectional dependence detected
MMR	Southeast Asia	χ²(11) = 2030.06***	F(1,10) = 2.666	2.965**	Heteroscedasticity and cross-sectional dependence detected; no evidence of serial correlation
NMR	South Asia	χ²(8) = 142.24***	F(1,7) = 104.017***	2.528*	Heteroscedasticity, serial correlation and cross-sectional dependence detected
NMR	Southeast Asia	χ²(11) = 1068.94***	F(1,10) = 26.120***	1.166	Heteroscedasticity and serial correlation detected; no evidence of cross-sectional dependence
